# Parietal function in good and poor readers

**DOI:** 10.1186/1744-9081-2-26

**Published:** 2006-08-01

**Authors:** Robin Laycock, Sheila G Crewther, Patricia M Kiely, David P Crewther

**Affiliations:** 1 School of Psychological Science, La Trobe University, Bundoora, Victoria, 3086, Australia; 2 Brain Sciences Institute, Swinburne University of Technology, 400 Burwood Rd, Hawthorn, Victoria, 3122, Australia

## Abstract

**Background:**

While there are many psychophysical reports of impaired magnocellular pathway function in developmental dyslexia (DD), few have investigated parietal function, the major projection of this pathway, in good and poor readers closely matched for nonverbal intelligence. In view of new feedforward-feedback theories of visual processing, impaired magnocellular function raises the question of whether all visually-driven functions or only those associated with parietal cortex functions are equally impaired and if so, whether parietal performance is more closely related to general ability levels than reading ability.

**Methods:**

Reading accuracy and performance on psychophysical tasks purported to selectively activate parietal cortex such as motion sensitivity, attentional tracking, and spatial localization was compared in 17 children with DD, 16 younger reading-age matched (RA) control children, and 46 good readers of similar chronological-age (CA) divided into CA-HighIQ and a CA-LowIQ matched to DD group nonverbal IQ.

**Results:**

In the age-matched groups no significant differences were found between DD and CA controls on any of the tasks relating to parietal function, although performance of the DD group and their nonverbal IQ scores was always lower. As expected, CA and RA group comparisons indicated purported parietal functioning improves with age. No difference in performance was seen on any of the parietally driven tasks between the DD and age-nonverbal IQ matched groups, whereas performance differentiated the DD group from the age-matched, higher nonverbal IQ group on several such tasks. An unexpected statistical difference in performance between lower reading age (DD and RA children) and all higher reading age (CA) children was seen on a test of chromatic sensitivity, whereas when high and low nonverbal IQ normal readers were compared performance was not different

**Conclusion:**

The results indicate that performance on purported parietal functions improves with age and may be more associated with nonverbal mentation than reading accuracy. Performance on a cognitively demanding task, traditionally considered to rely on ventral stream functions, was more related to reading accuracy.

## Background

Developmental dyslexia (DD) is the common term used to describe a significant impairment in learning to read relative to age, IQ, and educational and socioeconomic opportunity. Experienced by 4–10% ofchildren [1], developmental dyslexia occurs in all cultures examined [2] and persists into adulthood where impairment is characterised by slow reading [3,4]. Although the deficit is generally not attributed to IQ or any identified gross sensory or neurological problems [cf. [5]], the most accepted hypotheses to explain its cause are based on impaired auditory and phonological awareness and to a lesser extent impaired visual processing.

The visual deficits in processing of luminance contrast and motion sensitivity were originally interpreted as evidence of an impairment in the transient visual system rather than in the sustained system [6]. This was subsequently reformulated in the 90s as the magnocellular or M-pathway deficit hypothesis [7,8], and more recently refined further as an impairment in the dorsal M-pathway to parietal cortex [9,10]. Although it has also long been considered difficult to define a pure ventral type task, such tasks are not often considered impaired in DD. However, it has also been suggested that there are other equally parsimonious explanations apart from the dorsal M-pathway deficit [11-14], with the M-deficit theory possibly relevant to a sub-group of dyslexics [15,16].

Traditionally, the M-pathway has been considered to contribute the vast majority of the visual information carried by the dorsal visual stream to the primate parietal cortex [17] and to areas associated with transient visual attentional functions [18]. The ventral visual stream has been considered to receive substantial input contributions from both the M- and P-pathways and to terminate in the object recognition areas of the infero-temporal cortex [19].

In particular, to date there have been few systematic efforts to determine if the visual deficits seen in DD are specific only to the M-pathway to the human MT/V5 complex associated with motion perception, or M-pathway to higher visual functions of parietal cortex specifically involved in transient visual attention, or to both the dorsal and the ventral streams via its significant M-input.

Several research groups have started to explore alternative explanations, with important investigations into the M-dorsal visual stream using tests of motion coherence, change detection, and visual search [8,9,20,21]. For example, change detection, which has been shown with fMRI to be mediated by the intra parietal sulcus [22] is poorly performed by children with developmental dyslexia compared with controls [21].

Whether these M-dorsal deficits extend into a general parietal impairment has not yet been established, however it has been suggested that deficits seen in magnocellular function and a range of non-magnocellular perceptual tasks in disabled readers is reflective of a parietal deficit [23]. In addition a recent review has outlined the data showing impairments in fast attention shifts, abnormalities in eye-movements, and similarities with mild unilateral neglect syndrome are consistent with parietal deficits in dyslexia [10]. It was stressed that questions remain concerning other parietal functions. The association between DD and attention has also been investigated in a number of studies [18,24-27] with Vidyasagar & Pammer interpreting their results on a visual search task for shape defined by form and colour as indicating that when "attentional resources were stretched", tasks based on purported P-type stimuli as well as M-type tasks were impaired in reading disabled children.

Whether such a classification of visual search tasks as optimal for M- or P-pathways is justifiable is now highly controversial. Functional imaging techniques show that superior parietal cortex is activated in visual search defined by motion, colour or the conjunction of motion and colour [28] prior to activation of temporal cortex [29]. Braddick, O'Brien, Wattam-Bell, Atkinson, and Turner [30] have shown that detection of form from motion differentially activates ventral temporal areas as well as MT/V5 and parietal areas. Seidemann, Poirson, Wandell and Newsome [31] and Wandell et al. [32] have also shown that in area MT of primates, colour signals influence behavioural responses in speeded judgement tasks.

More recent theories of visual information processing incorporate traditional views of M and P contributions to dorsal and ventral processing, and also assert that the latency of arrival of foveal magnocellular visual information in V1 and then to V5, is such that it is fed back into V1 prior to visual perception[33] This idea of the perceptual advantage to magnocellular information could be termed a 'magnocellular advantage'. The numerous reports of the importance of visual function in dyslexia raises questions about whether all visual functions, both those traditionally considered dorsal, but also those considered ventral should show deficits in DD. The specificity of magnocellular processing in dyslexia may also need reconceptualising with reports of "the third" visual pathway, the koniocellular pathway having direct inputs into V5 [34], and having involvement with the detection of motion [35]. Such new ideas complicate explanations of dorsal stream, or magnocellular impairments such that careful interpretation must be made when classifying visual processing tasks as specific to specific cortical or subcortical systems [36].

When considering visual processing and its relationship to reading ability, it is also important to determine the extent to which general intelligence is involved. Excluding an independent role for intelligence in visually driven parietal processing is pertinent to dyslexia research given the controversy surrounding definitions of developmental dyslexia relying on a discrepancy between performance on IQ tests and reading performance [37-41]. Brain imaging studies have confirmed the importance of this by demonstrating that tasks associated with general intelligence correlate with regional activity in parietal cortex among other areas [42,43].

Hulslander et al. [44] recently concluded that their sensory (visual and auditory) processing tasks did not contribute to reading above and beyond the contribution of IQ. So whilst Hulslander et al. could make conclusions about more general sensory processing, whether or not individual differences in visual processing specifically targeting visually driven parietal functioning can be associated with reading ability after controlling for general intelligence has not been established.

Thus the current study aimed to investigate several purported parietal functions as a means of assessing whether supposed impairment in earlier M-pathway functioning in dyslexia would lead to an impairment of all visually driven parietal functions receiving their visual projection via the dorsal stream and whether this was related to general nonverbal problem solving ability. This was achieved through assessment of nonverbal general intelligence and performance on a number of tasks including spatial localisation and attentive tracking, both of which have been shown to specifically activate parietal cortex [45,46]. Assessment of motion sensitivity and the functional integrity of the motion areas in the M-dominated dorsal stream to area MT/V5 [47] was also made. A task involving hue discrimination, which has previously been considered to require ventral temporal lobe function, based on the relative "colour blindness" of M type neurons of retinal ganglion cells and the lateral geniculate nucleus [48], was also included to assess whether children with DD show generalized impaired performance possibly related to attentional demands of a time limited comparative visual and semantic task or to a generalized 'loss of concentration' under such test conditions [14,49,50].

## Methods

### Participants

Seventy-nine school age children with normal or corrected to normal visual acuity and colour vision participated in this study. A developmental dyslexia group (DD) comprised 17 children with a chronological reading age lag greater than two years [51] and Raven's Coloured Progressive Matrices non-verbal intelligence score within one standard deviation of the mean. Forty-six good readers formed a chronological-age control group (CA), and 16 younger good readers formed a reading-age control group (RA). Table 1 provides a summary of chronological age, reading age and mentation score for the three groups. Some children were tested in their schools, while others participated in the study as part of a specialist camp for children with reading difficulties.

**Table 1:** Mean and median chronological age, non-verbal intelligence and reading age for the three groups compared

**Group**	**No**.	**Chronological Age**		**Non-Verbal Mentation **(Ravens IQ)		**Reading Age**	
		
		**M**	**SE**	**M**	**SE**	**M**	**SE**
DD	17	11.7	0.4	95*	2	7.6**	0.3
CA	46	11.5	0.1	105	2	12.8	0.2
RA	16	8.1	0.1	106	3	8.5**	0.4

DD = developmental dyslexia; CA = Chronological age matched; RA = reading age matched.

Symbols denote significantly different from CA group.

(* = p < .01; ** = p < .0001).

The younger normally reading group (RA) was included to determine if parietal function is better predicted by chronological age, reading age or non-verbal intelligence. The use of two age groups of normal readers allows testing of the proposition that magnocellular functions continue to improve until 11–12 years of age [52-54]. The older normal readers would be expected to show better performance on purported dorsal/parietal tasks than the younger children, providing evidence for the continued development of magnocellular, or dorsally mediated, visual performance.

The current study had approval from the La Trobe University Human Ethics committee, schools, the Victorian Government Department of Education, and informed parental or guardian consent prior to any child's participation.

### Materials

#### Reading and non verbal mentation assessment

The reading age of 45 children from one school had been assessed using timed comprehension ability on the Reading Progress Test [55] prior to initiation of these experiments. As good comprehension is highly correlated with good decoding skills, a score above the 50^th ^percentile on the Reading Progress Test (which is a well-accepted measure in Victorian schools of reading comprehension skills) was deemed to be an adequate measure of good readers. Those who read below the expected level for their chronological age on this test, and all other participants in the study, completed the Neale Test of Reading Analysis Revised [56]. Of the 46 children in the CA group, 45 completed only the Reading Progress Test. All other children in the study completed the Neale.

Non-verbal intelligence was measured with the Coloured Progressive Matrices Test [57], for which different tests within the adult version have previously been shown by fMRI to induce brain activity across most of the cerebral hemispheres [58] suggesting that it is a good overall measure of nonverbal brain processing.

#### Stimuli and tasks

All visual tasks were completed on iMac computers (Apple Computer) equated for luminance and contrast settings. Participants were seated 57 cm from the screen (refresh rate was 117 Hz for most tasks, unless otherwise specified). Tasks were coded in VPixx (VPixx Technologies) using two alternate forced choice (2AFC) PEST procedures.

##### Motion sensitivity

In the Motion Coherence Detection task (see Additional file 1) a design similar to that described by Stein and colleagues [9,59] was used. Two identical adjacent rectangles (9° × 15° at 57 cm) were presented. Each rectangle contained 300 moving dots (each dot subtending 0.18°) and was presented for a trial duration of 2 sec (Stein's group have previously used stimulus intervals of 1.5 sec and 2.3 sec [9,60]). In one of the rectangles a varying proportion of the dots, beginning with 50%, moved coherently, in a horizontal direction, with a triangle wave motion (4 deg/s, amplitude 4°, temporal frequency 1 Hz), while the remainder of the dots moved in random directions with constant speed. The main difference between the current task and versions used by Stein's group is that in the current study dots did not have a limited lifetime restriction. Participants were required to indicate in which panel coherent motion was present. The number of coherently moving dots was gradually reduced under a PEST protocol.

A custom designed Apparent Motion 2-dot/4-dot Detection task was also utilised (see Additional file 2), and consisted of four circular patches ('dots') subtending 1° in diameter (at 57 cm) flashed on the screen in the four corners of an illusory square of size 6° × 6°. Participants were requested to indicate whether they perceived all four dots flashing on and off simultaneously (4-dot appearance; the percept appears for a variable amount of time, followed by a blank inter-stimulus interval of triple the stimulus presentation time); or two dots always present on the screen alternately vertically switching (i.e., top left and bottom right alternating with top right and bottom left; each percept appears for a variable amount of time, followed by a blank inter-stimulus interval of equal time). In this second condition the apparent motion appears as dots moving up and down along two separate sides of the illusory square (2-dot motion). In the 4-dot condition, a longer ISI was selected (three times the percept duration) in order that a full cycle of the stimulus was equal to the time of a full cycle of the 2-dot stimulus. Trial duration was 2 seconds to correspond with the motion coherence task, and the minimum percept exposure time at which the two stimuli (2-dot versus 4-dot) could be discriminated was estimated using a PEST procedure.

##### Spatial localization

Two custom designed tasks of spatial localization were used. The first, the Spatial Misalignment task (see Additional file 3), was based on that of Hess and Holliday [61], having three vertically aligned 'gabor' patches. In each trial, one gabor was misaligned horizontally with respect to the remaining two. Subjects were required to indicate in a two alternate forced choice (2AFC) design the direction of horizontal misalignment (i.e. the left or right side of the vertical fixation plane) of the gabor that did not align with the other two, but were not required to specify which gabor was misaligned. The gabor stimulus consisted of a sinusoidal grating with a spatial frequency of 2 cycles degree^-1 ^and Michelson contrast of 0.5, enveloped by a circular Gaussian envelope (SD 0.5 degrees). Stimuli were presented for 1 sec, as piloting showed this to be sufficient for adequate performance, on a computer monitor of background luminance approximately 50 cd/m^2^. Screen resolution was set at 1024 × 768, with a refresh rate of 75 Hz. Gabors subtended approximately 2° in width and height and were separated by 4° of visual angle (for a viewing distance of 57 cm). The minimum offset under which misalignment at each of the three positions could be reliably discriminated was determined with a separate PEST procedure for each gabor, and interwoven into one testing session.

The second spatial localization measure, the Length Discrimination task (see Additional file 4) consisted of two parallel horizontal black rectangles subtending 1° in height with one of varying length (the reference subtending 10° horizontally), randomly offset horizontally at both ends. They were presented on a grey background for 8 frames (68 ms), then followed by a rectangular mask (15° × 6°) consisting of visual noise (random dot with a grain of 2 pixels and 50% contrast). A short exposure time was set so participants would not have time to make eye-movements between the bars. The two bars were separated vertically by a gap subtending 1° at 57 cm in the centre of which was a fixation point. The threshold was the minimum difference in length at which subjects could reliably identify the longer bar with 80% confidence.

##### Shifting attention

**Figure 1:**
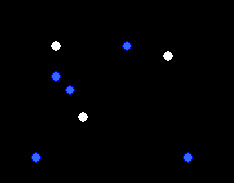
An example of the Attentive Tracking task in which there are three target white balls amongst, in this case, five blue distracter balls. The three target balls then change colour to blue and must be tracked by the participant.

A custom designed sustained Attentive Tracking task (see Figure 1) was based on the "FINST" task of Pylyshyn and Storm [62]. The task requires fixation on a central point while three white target balls and a variable number of blue distracter balls move randomly around the screen, bouncing off the edges of the screen and off each other. After three seconds, the white balls change colour to become indistinguishable distracter balls. After 7 more seconds, requiring sustained tracking, the balls abruptly stopped and participants were required to click on the balls that were originally white by using a mouse click on the computer monitor. Two consecutive correct responses resulted in an additional distracter ball being added, whilst a single incorrect response resulted in the removal of one distracter ball. A two down, one up staircase PEST routine was used to determine the threshold number of balls participants could reliably track. This task was coded in Authorware Professional (Macromedia) and Code Warrior (Metrowerks). Fixation of the screen was monitored by the experimenter. RA control children proved unable to maintain sustained fixation, even with the simplest 3 ball tracking task and were thus excluded from this task.

##### Ventral stream function

Ventral stream function was targeted with a custom designed Hue Discrimination task (see Additional file 5) which required 2AFC identification of two coloured parallel rectangular bars subtending 10° in width and 1° in height, with hue lying on a red/green radiometrically isoluminant continuum. The bars were presented for 684 ms on the screen and followed by a mask. Subjects were required to indicate in a 2AFC design which line was 'redder', with testing only commencing once understanding had been gained through familiarisation. Training participants in this way would have revealed any colour blindness and resulted in exclusion from the task. This colour comparison was expected to be primarily processed in ventral temporal cortex which is considered to receive M and P projections [63]. Previous primate research has demonstrated that the P- and koniocellular pathways, rather than the M-pathway, are predominantly involved in colour discrimination [64]. Chromatic threshold was established with a PEST procedure and was defined as the least variation from yellow that subjects could reliably distinguish at an 80% confidence level.

### Data analysis

Data was examined for violations to the assumptions of normality and homogeneity of variance and it was found that data for the different variables were distributed differently within the groups compared. Transformations were not considered appropriate and thus non-parametric tests were utilised (Mann-Whitney U for group comparisons, and the Spearman Rank correlation), avoiding the assumptions and requirements associated with parametric tests.

Although these tasks (excluding the Hue Discrimination task) have been primarily designed to activate parietal cortex, they were not expected to be directly related to each other [see [65] for discussion of this point]. In order to ascertain whether all tasks were independent, a correlation matrix was run. Following Bonferroni corrections for type 1 error rate, no significant correlations were established between the different tasks, suggesting that family-wise error rates were not expected to be important when running multiple comparisons between groups.

## Results

### Descriptive statistics

Although the DD group were selected with a commonly used criterion (a two-year reading lag, and nonverbal intelligence within normal range), as can be seen from Table 1, the current sample demonstrated a significantly lower nonverbal IQ score compared with the CA control group (*p *< .001). Thus we could not be sure that any difference between groups in parietal performance would relate to reading ability exclusively. This major confound served to justify splitting the normally reading control group into three groups of performers of similar numbers on the same nonverbal IQ test in order to subsequently examine the role of nonverbal IQ in parietal functioning.

As is seen in Table 2, The bottom third from the CA group comprised the CA[low IQ] group showing normal reading and similar nonverbal IQ to the DD group. The top third on the Ravens task comprised the CA[high IQ] group showing normal reading and high nonverbal IQ. As expected, based on group selection, the DD group and the CA[low IQ] groups did not differ on the Ravens nonverbal intelligence measure (*Z *= -0.2, *p *= .83).

**Table 2:** Mean chronological age, non-verbal intelligence and reading age for the four groups compared

Group	No.	**Chronological Age**		**Non-Verbal Mentation **(Ravens IQ)		**Reading age**	
		
		**M**	**SE**	**M**	**SE**	**M**	**SE**
DD	17	11.7	0.4	95	2	7.6	0.3
CA[L]	16	11.4	0.2	93	1	12.3**	0.3
CA[H]	16	11.8	0.2	118**	1	13.4**	0.5
RA	16	8.1	0.1	106*	3	8.5	0.4

DD = developmental dyslexia; CA[L] = Chronological age matched, low IQ; CA[H] = Chronological age matched, high IQ; RA = reading age matched.

Symbols denote significantly different from DD group.

(* = p < .01; ** = p < .0001).

We therefore decided to present the comparisons for the entire CA group with the other groups, on all visual tasks, and then to examine the influence of nonverbal intelligence in subsequent comparisons.

### Comparison of DD, CA and RA groups

#### Motion sensitivity

**Figure 2:**
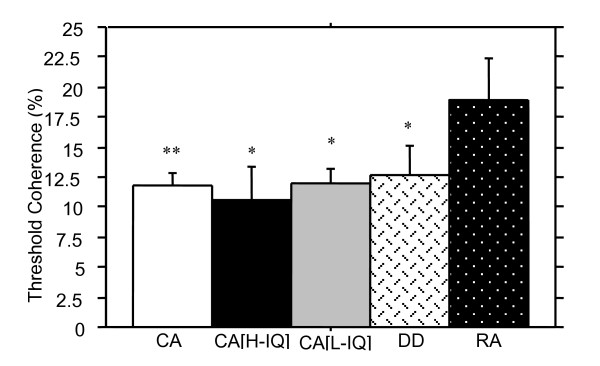
Motion coherence thresholds (measured as percentage of coherently moving dots at threshold) (+ SE) for all groups. The CA control group showed the best performance (lowest percentage of coherent dots) but did not differ significantly from performance of the DD group. Worst performance was seen for the chronologically youngest group, the RA controls, and no difference in performance was seen between the high and low intelligence control groups, suggesting chronological age is a better predictor of motion coherence threshold than reading ability or intelligence. (Symbols denotes significantly different from RA group, * p < .05; ** p < .01).

Figure 2 shows the mean scores for the motion coherence detection task for all groups. Not surprisingly the CA control children showed significantly lower thresholds for motion sensitivity than the younger RA matched control children, *Z *= -2.4, *p *< .01. The DD group's mean threshold for motion coherence detection (*M *= 12.59, *SD *= 10.41) was not significantly different from that of the CA control group (*M *= 11.74, *SD *= 7.60), *Z *= -0.2, *p *= .43. However, DD children did show significantly lower thresholds on the motion coherence detection task when compared with the RA children *Z *= -2.1, *p *< .05.

**Figure 3:**
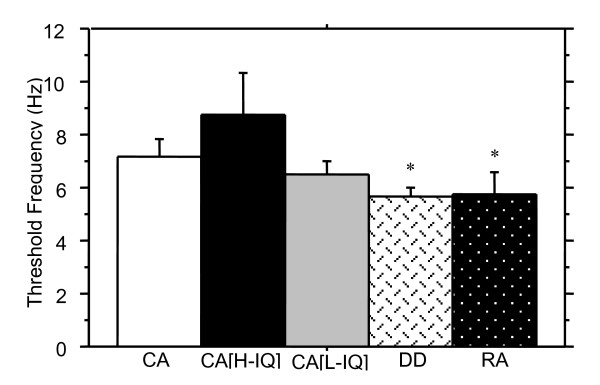
Threshold discrimination of 2-dot motion versus 4-dot appearance (+ SE) for all groups compared is shown. The best performance (highest temporal frequency at which the two percepts could be discriminated) was shown by the CA [high IQ] group. The only significant difference was seen between the DD and CA [high IQ] groups, indicating that on this task nonverbal intelligence is the better predictor of performance, although the high and low IQ normal readers' performance was not significantly different. (* denotes significantly different from CA[H], p < .05).

The scores for children on the second motion sensitivity task, the Apparent motion 2-dot/4-dot detection task can be seen in Figure 3. Although CA children showed higher fusion thresholds than the RA and DD groups, there were no statistically significant differences between CA and RA groups, *Z *= -1.4, *p *= .08, CA and DD groups, *Z *= -0.9, *p *= .17, nor between the DD group and RA control group, *Z *= -0.5, *p *= .61.

#### Spatial localization

**Figure 4:**
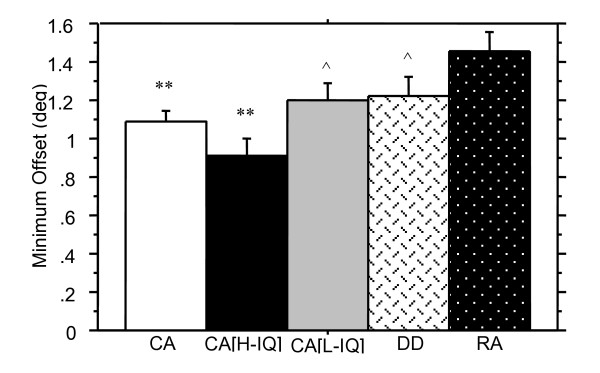
Spatial localization thresholds (minimum horizontal offset of the misaligned gabor) are shown in degrees (+ SE) for the average performance across each of the three gabor positions. The older CA [high IQ] good readers performed best, with the DD and CA [low IQ] groups performing comparably, indicating that nonverbal IQ is a more significant predictor than reading ability for this task. Worst performance was seen by the RA group. Comparisons between CA and RA children indicates a developmental trend in spatial localization. (** denotes significantly different from RA group, p < .01; ^denotes significantly different from CA[H] group, p < .05).

Figure 4 shows average thresholds in the Spatial Misalignment task for all three gabors averaged together for each group of children. Table 3 summarises the Mann-Whitney results for all comparisons made. The average threshold across all three gabors on the Spatial Misalignment task differentiated CA and RA children. Better judgement of spatial localization on each individual gabor was also seen by the CA group when compared with the RA group.

**Table 3:** Mann-Whitney U results for the Spatial Misalignment task.

	CA				RA			
	
	average	top	middle	bottom	average	Top	middle	bottom
DD	*Z *= -1.1	*Z *= -0.3	*Z *= -1.5	*Z *= -0.6	*Z *= -1.5	*Z *= -1.6	*Z *= -0.9	*Z *= -1.0
	*p *= .15	*p *= .38	*p *= .07	*p *= .29	*p *= .14	*p *= .10	*p *= .38	*p *= .34
RA	*Z *= -2.8	*Z *= -2.3	*Z *= -2.8	*Z *= -1.7				
	*p *< .01	*p *< .01	*p *< .01	*p *< .05				

The DD group had higher average thresholds on the Spatial Misalignment task than did the CA controls, indicating poorer performance, but the difference was not significant. Variance in performance between these groups appears to arise primarily from the middle gabor. Thresholds for the top and bottom gabors were not significantly different between DD and CA groups, and although the DD group has a higher threshold than the CA group for the middle gabor, this also is not statistically significant.

DD children showed no significant differences in performance on the Spatial Misalignment task compared to the RA group for the average threshold across all three gabor patches nor was there a significant difference between groups for threshold scores on each of the three gabors individually.

**Figure 5:**
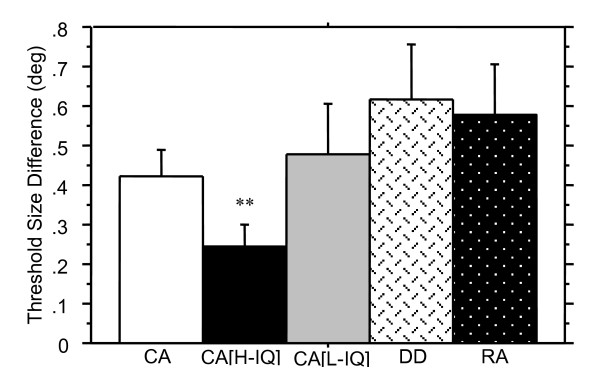
Length discrimination thresholds (in degrees of visual angle + SE) are shown for the three groups. A lower threshold indicates better performance. The DD group showed inferior ability to perform this task, differing significantly from the CA [high IQ] group, and (non-significantly) from the CA low IQ group. Neither age nor reading performance proved good predictors of performance, whilst nonverbal intelligence appears more important. (** denotes significantly different from DD group, p < .01).

Thresholds on the Length Discrimination task for all groups are shown in Figure 5. There was no significant difference in threshold scores of length judgement between the CA and RA control groups, *Z *= -1.1, *p *= .13, or the DD and RA groups, *Z *= -0.5, *p *= .59. Consistent with the prior Spatial Misalignment Task, Figure 5 indicates that the DD group's average threshold for the Length Discrimination task was higher than for the CA control group but the difference was not significant, *Z *= -1.5, *p *= .07.

#### Shifting attention

**Figure 6:**
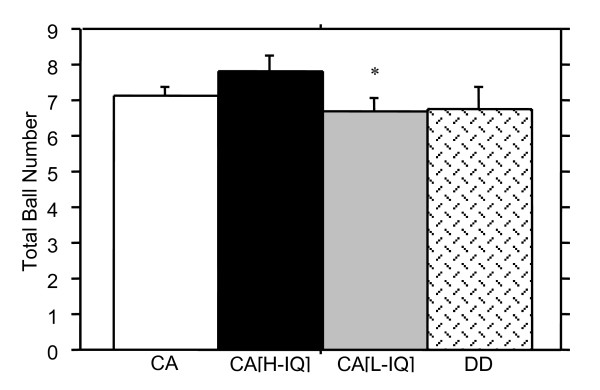
Attentive tracking thresholds (+ SE) are shown as the total number of balls (i.e. the number tracked, plus distracters). Results for the two older groups only are shown, as very few of the RA group were able to complete the task. Little difference was shown in performance between the DD and any of the CA groups. Whilst the DD and CA [low IQ] groups performed equally, the CA [high IQ] group outperformed both groups indicating that nonverbal IQ rather than reading ability is a better predictor of performance. (* denotes significantly different from CA[high IQ] group, p < .05).

The threshold for number of balls tracked in the Attentive Tracking task for DD and CA children are shown in Figure 6. This task was not administered to many of the RA group as it proved too difficult for most and thus the results for only two groups were analysed. Results clearly indicate that DD readers were not deficient on this task compared with the CA group, *Z *= -0.6, *p *= .29.

#### Ventral stream (P) function

**Figure 7:**
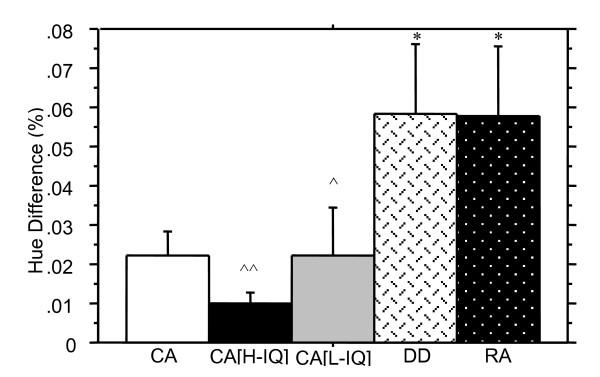
Hue discrimination thresholds (+ SE) for the identification of the "redder" of two bars are shown as the percentage difference. Better performance is indicated by a lower threshold. The DD and RA groups performed comparably, with both performing significantly worse than the CA group indicating that reading ability is a better predictor of performance on this task than age. The CA [high IQ] group clearly showed the best performance. Whilst the CA [low IQ] group showed marginally poorer performance, this group performed significantly better than both the DD and RA children, again indicating that reading ability rather than IQ is likely to contribute more to performance of this ventral stream task. (* denotes significantly different from CA group, p < .05; ^denotes significantly different from DD group, p < .05; ^^ p < .01).

Results for the Hue Discrimination task are shown in Figure 7, and indicate that the RA group required a substantially larger difference in spectral intensity for discrimination of the 'redder' bar in the Hue Discrimination task when compared to the CA group, *Z *= -2.3, *p *< .05. Significant differences were also established between the DD and CA groups on this task, despite the large standard error for the DD group, *Z *= -2.1, *p *< .05. No significant differences in performance were seen between the DD and RA groups *Z *= -0.1, *p *= .94.

#### Trends in DD performance

None of the five tasks purported to require parietal functioning (motion coherence detection, apparent motion 2-dot/4-dot detection, spatial misalignment task, length discrimination task, and attentive tracking task) showed predicted significant differences between dyslexic and age-controlled children. Nevertheless, Figures 2, 3, 4, 5, 6 illustrate superior mean scores on all tasks by the entire CA group (see Table 4). A sign test was thus run, with results suggesting that such a pattern of findings could not be expected if there was truly no difference between groups (*p *< .05). In addition, it is worth noting that performance on the Hue Discrimination task demonstrated a similar trend whereby the CA group performed better than the DD group.

**Table 4:** Mean scores of DD and CA groups on tasks expected to show group differences

Test	Group		Direction of Difference*
		
	DD	CA	
Motion Coherence Detection	12.6	11.7	+
Apparent Motion 2Dot/4Dot Detection	5.7	7.2	+
Spatial Misalignment	1.2	1.7	+
Length Discrimination	0.6	0.4	+
Ball Tracking	6.8	7.1	+

* '+' indicates superior performance by the CA group over the DD group.

### Comparisons based on nonverbal IQ and reading

In the current sample, despite selecting dyslexics on the basis of having nonverbal intelligence in the normal range, the DD group showed significantly reduced nonverbal intelligence scores compared with age matched controls (*p *< .001). Thus we could not be sure that the trend for impaired parietal performance was a reflection of reading ability exclusively. The subsequent results show comparisons between the DD, RA and high-IQ and low-IQ control groups in order to examine the role of nonverbal IQ in parietal functioning. (For comparisons of performance for low-, high- and medium-IQ groups, which typically did not differ from the trend borne out in the current data showing low and high groups only, see Additional file 6)

#### Comparisons between DD and normal readers with low nonverbal IQ

For comparisons between the DD and CA[low IQ] groups, where nonverbal intelligence was held constant, and reading ability was the differentiating variable, no significant differences were found on the Motion Coherence task, *Z *= -0.3, *p *= .81, the 2-Dot 4-Dot motion task, *Z *= -1.2, *p *= .23, the Spatial Misalignment task, (see Table 5 for results of individual gabors, and averaged across all gabors), the Length Discrimination task, *Z *= -0.8, *p *= .42, nor on the Attentive Tracking task, *Z *= -0.2, *p *= .87. Performance of the CA[low IQ] group on the Hue discrimination task was significantly better than that of the DD group, *Z *= -2.5, *p *< .05.

**Table 5:** Mann-Whitney U results for the Spatial Misalignment task.

	CA [low IQ]				CA [high IQ]			
	
	average	top	middle	bottom	average	top	middle	bottom
DD	*Z *=-0.1	*Z *= -0.5	*Z *= -1.3	*Z *= -0.3	*Z *= -2.1	*Z *= -1.3	*Z *= -2.2	*Z *= -1.3
	*p *= .96	*p *= .59	*p *= .22	*p *= .79	*p *< .05	*p *= .11	*p *< .05	*p *= .10
CA [high IQ]	*Z *= -2.1	*Z *= -1.9	*Z *= -1.4	*Z *= -1.8				
	*p *< .05	*p *< .05	*p *= .08	*p *< .05				

#### Comparisons between the DD and normal readers with high nonverbal IQ

When comparing the DD group with a group superior in reading ability and nonverbal intelligence (CA[high IQ] group), significant differences were established on several tests. Whilst performance on the Motion Coherence task was comparable between groups, *Z *= -1.0, *p *= .16, the other motion sensitivity measure, the 2-Dot 4-Dot motion task differentiated significantly between the DD and the CA[high IQ] group, *Z *= -1.9, *p *< .05.

The CA[high IQ] group performed significantly better than the DD group on the Spatial Misalignment task, however only spatial localisation performance on the middle gabor demonstrated a significant difference (see Table 5). Similarly, the high nonverbal IQ group of normal readers showed significantly better performance on the Length Discrimination task, *Z *= -2.5, *p *< .01.

On the Attentive Tracking task no significant difference in performance was found despite the DD group showing a lower mean, *Z *= -1.4, *p *= .08, whilst the Hue Discrimination task also showed inferior performance by the DD group, *Z *= -2.9, *p *< .01.

#### Comparisons between normal readers with high and low nonverbal IQ

Comparisons between the two groups, with reading ability held constant, and nonverbal intelligence differing showed some significant differences. No significant differences were found on the Motion Coherence task, *Z *= -1.6, *p *= .06, nor on the 2-Dot 4-Dot motion task, *Z *= -0.7, *p *= .25. However, the high nonverbal IQ group showed superior overall performance on the Spatial Misalignment task, and on two of the gabors individually (see Table 5). The high nonverbal IQ group also showed superior performance on the Length Discrimination task, but this difference was not significant, *Z *= -1.3, *p *= .10, whereas in the Attentive Tracking task the high nonverbal IQ group did demonstrate superior performance, *Z *= -1.9, *p *= .05. On the Hue Discrimination task, no difference was found between the high and low nonverbal IQ groups, *Z *= -0.7, *p *= .46, despite the high nonverbal IQ group showing a lower (superior) mean.

## Discussion

We assessed reading accuracy, nonverbal IQ, and performance of 79 school age children on a range of visual tasks in. The majority of the visual tasks have been shown by fMRI to primarily activate the parietal cortex; while we also included a purported measure of ventral function. On the basis of the M-deficit hypothesis for children with dyslexia [8] it was predicted that if the dorsal visual pathway was impaired then only the visual functions of the parietal cortex – which receives predominantly M-pathway projections – and not the ventral, P-type, hue discrimination task would be impaired.

However this hypothesis relies on the traditional feedforward view of the visual system. Whereas, if we invoke a feedforward/feedback model of visual processing, then impairments in cognitively demanding ventral processing would also be expected if dyslexia is associated with the outcomes of a 'magnocellular disadvantage', meaning that the magnocellular contributions do not retroinject back intoV1 in time for the more fine detailed parvicellular processing through the ventral stream. This turned out to be the case, with the only task significantly differentiating between the children with DD and the same chronological age normal readers being the hue discrimination task. We return to this later in the discussion.

There was, nevertheless, a generalized trend for the DD group to show impaired performance on all the parietal tasks. However, given that the dyslexic group mean was below the 50^th ^percentile in nonverbal intelligence (although the range is 16^th ^– 84^th ^percentile), attributing this trend in impaired parietal performance to reading ability alone was considered premature, and a further split of the CA group into three nonverbal intelligence groups was made.

### Development of dorsal and ventral visual function and developmental dyslexia

The concept of development in all visually mediated tasks with increasing age received support from the results which indicated that younger (RA) normally reading children showed inferior performance compared with the older (CA) normally reading children on all tasks. The performance of the older children was significantly better on all tasks except two measures – the apparent motion 2-dot/4-dot detection task which showed a trend toward significance, and the length discrimination task – whether believed to target specifically dorsal-M functioning, general parietal cortex functioning, or ventral (P) functioning when compared to older (CA) normally reading children. This has previously been suggested by Barnard et al. [52] and Crewther et al. [54] who found psychophysical and electrophysiological evidence for developmental improvement in magnocellular functions with age up to 10–12 years, while Gordon and McCulloch [66] interpreted their results as electrophysiological evidence for parvocellular but not magnocellular until development until puberty.

The finding that younger normally reading children performed similarly to the DD group on all tasks except the Motion Coherence task, suggests that children with developmental dyslexia show symptoms of a developmental delay in dorsal/parietal functions. However the great variation in nonverbal intelligence of the groups potentially complicates this interpretation. The Motion Coherence task, where DD children showed superior performance compared with the younger chronological age but reading-age matched children, suggests that motion coherence may not be targeting similar dorsal/parietal areas as the other tasks. Indeed, the use of motion coherence as a measure of magnocellular sensitivity has recently come under scrutiny [36]. Equally, this result may indicate that motion coherence detection is a better indicator of visual processing that distinguishes lower reading ability from developmental dyslexia.

Unexpectedly, statistical analysis of the two measures of motion sensitivity did not reveal the significant difference in performance between the good and poor reading ability groups expected from previous studies [60,67]. However, it is noteworthy that the statistical but not clinically significant difference in mean thresholds of coherent dots recorded in Cornelissen's study for normal children (11.1%) and those with dyslexia (13.7%) did not differ greatly from the numbers in the current study (11.7% and 12.6%, respectively). Secondly, our longer durations increased the likelihood of all children having time to concentrate on the task [14], and possibly decreased the probability of discrimination between good and poor readers. Similarly, the two spatial localization tasks here failed to support the hypothesis that the DD group would show a deficit in spatial localization, a function of parietal cortex.

Clinical neuroscience has long recognised the role of parietal cortex in static attention and its activation prior to the shifts in attention which precede eye movements [26,68,69]. Thus as performance of the DD and CA control children did not differ on the attentive tracking task, which is considered a measure of shifting of attention, it is unlikely that dyslexia is primarily associated with deficits in the parietal cortex *per se*, though it may be associated with rate of processing for parietal tasks. DD and good readers apparently shift attention equally well between an increasing number of target balls presented at the reasonably slow rate of ball movement, with few unexpected rapid changes in direction as used here.

By comparison, the Hue Discrimination task, a purported P pathway driven ventral stream processing task, differentiated between the younger and older normal readers, and, unexpectedly, between the CA and DD groups. Possibly, however, this task requires a greater level of semantic or verbally categorised visual discrimination than the other tasks, rather than being a reflection of visual maturation. Interestingly, Newsome and his colleagues have recently shown electrophysiologically, that colour information aids rapid speed judgements in primate middle temporal motion region [31,32]. As the Hue Discrimination task was the only task that significantly discriminated the DD from the whole CA group this result may have implications for understanding of the temporal sequence of activation of visual functioning in dorsal and ventral areas in good and poor readers. Such a significant difference in Hue Discrimination between the DD group and the children with matched nonverbal IQ scores would not be surprising if the feedforward-feedback model of visual processing of Bullier is correct and a 'magnocellular latency advantage' is necessary to rapidly activate attention mechanisms in parietal cortex prior to conscious ventral processing. Bullier's model would predict that a longer latency for the magnocellular pathway to arrive in V1 travel to V5 and back to V1 would make for less efficient ventral processing. It may then be hypothesised that the rate of firing would be less coherent in dyslexia – a 'magnocellular disadvantage'. Further discussion of this will be made below.

### Is nonverbal intelligence or reading ability more related to performance on visually driven parietal function?

Comparisons between children with DD and children with matched (lower) nonverbal IQ but superior reading ability showed no difference in performance on purportedly visually driven parietal tasks. This observation leads to the proposition that nonverbal intelligence, not reading ability, is more important in determining performance. On the other hand, comparisons between, firstly, children with DD and high nonverbal IQ good readers, and secondly, low and high nonverbal IQ good reading children showed significant differences in performance on a range of tasks expected to target visually driven parietal cortex. This observation then, leads to a similar conclusion that nonverbal IQ seems to be an important determinant in performance on these tasks. This study may have implications for definitions of developmental dyslexia using a discrepancy based discrimination, especially a nonverbal IQ discrepancy [37] and seems to support the large body of literature highlighting the problems of relying on such a criterion [38-40,70].

### A role for magnocellular processing in poor readers

Despite difficulties associated with controlling for nonverbal IQ, certain conclusions can be made from the current data about visual processing in poor readers. Even when the properties of stimuli were expected to selectively target the ventral stream, for which there are only a few reports of impairment [16,26], children with lower reading ability (DD and RA groups) performed worse than all good reading groups. In fact, reading accuracy appears to be a more important predictor of hue discrimination than nonverbal IQ. This is supported by the finding that low and high nonverbal IQ good readers could not be distinguished on this task. In fact, this task was the only one to present a clear pattern of results of distinguishing between reading ability. On the surface, it may be concluded that ventral, or parvocellular, processing is impaired in the DD group. This conclusion may be premature however.

Alternatively, the current results, in which a trend for reduced performance was seen in the M-dorsal, parietal, and the ventral tasks, appear consistent with Amitay et al [23] who also found impairment in a range of magnocellular and non-magnocellular perceptual tasks. Amitay et al. proposed that a specific parietal lobe impairment may explain such data. A specific role for magnocellular processing in such a parietal deficit, however, was ruled out due to anatomical and physiological studies highlighting that the correspondence between the subcortical and cortical visual pathways is only partial. These studies have pointed out that the M- and P-pathways remain segregated through the LGN, however beyond V1 there is a large amount of interconnections between pathways, and M-, P- and K-contributions are made through the dorsal stream into parietal regions. Parietal attention mechanisms may indeed help explain both sets of data, however, we prefer to highlight the role of feedforward/feedback magnocellular processing through the dorsal stream as suggested by more recent models of visual processing [33].

Discriminating between 'red' and 'green' is a categorical task which would be ideally suited to the ventral stream. However, at threshold, the task becomes much harder. Discrimination must be made between two similarly coloured bars, not easily categorised as distinctly different colours. It is postulated that in this case, extra attentional resources may be recruited from parietal cortex. However, as parietal cortex is not well equipped for colour processing, it might be expected that the load on attentional resources is high, and thus more processing time would be required. If children with a reading disability have a visually based impairment of transient attention, then one would expect just this type of task, where rapid activation of parietal areas are required to assist in traditional P-type processing through the ventral stream [33], to show the pattern of results found here. This type of explanation seems congruent with Vidyasagar and Pammer who concluded that when attentional resources were stretched, all visual performance would be impaired in dyslexia.

It is therefore postulated that the magnocellular advantage, involving a rapid feedforward-feedback loop through the dorsal stream, and arriving back in V1 in time for the initial arrival of parvocellular injections, is an essential feature for object recognition and discrimination, and is potentially the best explanation for reading fluency. Further investigations would be required to ascertain the applicability of this theory.

## Conclusion

This study raises three distinct yet related conclusions. Firstly, whilst reading accuracy does not appear to be significantly related to visually driven parietal functioning, accurate reading ability may be related to rapid visual processing under conditions requiring cognitively demanding (ventral) processing. It is hypothesised that even in such tasks, expected to be more reliant on parvocellular processing, the necessary recruitment of parietal attention mechanisms may highlight an impairment in children with dyslexia. This appears to support newer feedback models of visual processing. Secondly, parietal functioning improves with age, as does ventral functioning, providing evidence for development in all visually mediated tasks. This conclusion maybe tempered by the possibility of the purported ventral stream task not relying on purely P type functioning.

Thirdly, traditional definitions of dyslexia do not appear appropriate in determining such visual functioning. On the one hand, visually driven parietal functioning appears more related to nonverbal intelligence, and on the other hand, where visual driven information is associated with reading ability, nonverbal intelligence is less important. This may suggest that categorisation of children as having a 'specific reading impairment' may be less applicable, than is a classification of 'poor reading' for developmental dyslexia type research.

Dyslexia research must therefore seek to explore if and how more general learning disabilities and garden variety poor readers are different from classical definitions of dyslexia with further investigations into the role of transient visual processing in the ability to rapidly decode the words on a page.

## Competing interests

The author(s) declare that they have no competing interests.

## Authors' contributions

RL participated in the design and co-ordination of the study, data collection, data analysis and drafted the manuscript. SC participated in the design of the study, data collection and preparation of the manuscript. PK participated in data collection and helped to draft the manuscript. DC designed the stimuli, participated in data collection, data analysis and helped with the manuscript.

## Supplementary Material

Additional File 1 This demonstration of the Motion Coherence Detection task shows two panels with randomly moving dots. In one panel (left) a proportion of the total dots (50% in this example) are moving coherently in the same direction. Click here for file

Additional File 2 This demonstration of the Apparent Motion 2-dot/4-dot Detection task shows first the 2-dot condition. Initially a slow frequency is shown whereby apparent motion is created, followed by a faster frequency closer to threshold where it is more difficult to discriminate conditions. Next the 4-dot condition is shown, firstly at a slow, and then at a faster frequency closer to threshold. Click here for file

Additional File 3 The Spatial Misalignment task is shown, in this example with the top Gabor misaligned towards the right side. Click here for file

Additional File 4 Length Discrimination in which participants must rapidly scan both ends of the lines and determine which line is longer. In this example the top rectangle is shown as longer, and then followed by a mask. Click here for file

Additional File 5 Two demonstrations of the Hue Discrimination task in which participants must determine which line is 'redder' is shown. Firstly, an easier presentation of red and green bars flowed by a mask. Secondly, two bars are shown which are closer together on the colour spectrum – in this example the top bar is 'redder'. Click here for file

Additional File 6 Supplementary results showing ANOVA tables, bar graphs, and post hoc results for non-verbal intelligence, motion sensitivity measures, spatial localisation measures, the shifting attention task, and the ventral stream function task. Click here for file
