# Supplementary material for: Parietal function in good and poor readers
Source: Behav Brain Funct. 2006 Aug 1;2:26. doi: 10.1186/1744-9081-2-26 (PMC1553455; doi:10.1186/1744-9081-2-26)
Supplement: Additional File 6 — Supplementary results showing ANOVA tables, bar graphs, and post hoc results for non-verbal intelligence, motion sensitivity measures, spatial localisation measures, the shifting attention task, and the ventral stream function task. [file 1744-9081-2-26-S6.doc]

2

5154.552

2577.276

186.984

<.0001

373.967

1.000

43

592.687

13.783

DF

Sum of Squares

Mean Square

F-Value

P-Value

Lambda

Power

L-IQ- M-IQ H-IQ

Residual

16

93.062

3.276

.819

14

104.000

2.112

.565

16

118.375

4.992

1.248

Count

Mean

Std. Dev.

Std. Err.

L-IQ

M-IQ

H-IQ

**Means Table for nonverbal IQ**

0

20

40

60

80

100

120

140

IQ

L-IQ

M-IQ

H-IQ

-10.938

2.740

<.0001

S

-25.312

2.647

<.0001

S

-14.375

2.740

<.0001

S

Mean Diff.

Crit. Diff.

P-Value

L-IQ, M-IQ

L-IQ, H-IQ

M-IQ, H-IQ

**POST-HOC TESTS**

**Fisher's PLSD for nonverbal IQ**

**L-IQ = low IQ group; M-IQ = medium IQ group; H-IQ = high IQ group;**

**S= significant**

**Significance Level: 5 %**

**Error Bars: ± 1 Standard Error(s)**

**Nonverbal Intelligence**

**ANOVA Table for Non verbal IQ**

2

41.879

20.939

.351

.7057

.703

.101

42

2502.215

59.577

DF

Sum of Squares

Mean Square

F-Value

P-Value

Lambda

Power

Residual

**Motion Sensitivity**

**Motion Coherence Detection task**

**ANOVA Table for Motion Coherence**

15

12.002

4.664

1.204

14

12.844

3.894

1.041

16

10.521

11.549

2.887

Count

Mean

Std. Dev.

Std. Err.

L-IQ

M-IQ

H-IQ

**Means Table for Motion Coherence**

0

2

4

6

8

10

12

14

16

18

Coherence threshold

L-IQ

M-IQ

H-IQ

-.842

5.788

.7706

1.482

5.598

.5961

2.323

5.701

.4155

Mean Diff.

Crit. Diff.

P-Value

**POST-HOC TESTS**

**Fisher's PLSD for Motion Coherence**

**Significance Level: 5 %**

L-IQ- M-IQ H-IQ

L-IQ, M-IQ

L-IQ, H-IQ

M-IQ, H-IQ

**L-IQ = low IQ group; M-IQ = medium IQ group; H-IQ = high IQ group**

**Error Bars: ± 1 Standard Error(s)**

L-IQ

M-IQ

H-IQ

2

64.607

32.304

1.418

.2533

2.836

.277

43

979.546

22.780

DF

Sum of Squares

Mean Square

F-Value

P-Value

Lambda

Power

Residual

**Apparent Motion 2-Dot/4-dot detection task**

**ANOVA Table for Apparent motion 2-dot/4-dot detection**

16

6.528

1.971

.493

14

6.051

5.190

1.387

16

8.761

6.170

1.543

Count

Mean

Std. Dev.

Std. Err.

L-IQ

M-IQ

H-IQ

**Means Table for Apparent motion 2-dot/4-dot detection**

0

2

4

6

8

10

12

Threshold Frequency

L-IQ

M-IQ

H-IQ

**Error Bars: ± 1 Standard Error(s)**

.477

3.523

.7859

-2.233

3.403

.1928

-2.710

3.523

.1281

Mean Diff.

Crit. Diff.

P-Value

**POST-HOC TESTS**

**Fisher's PLSD for Apparent motion 2-dot/4-dot detection**

**Significance Level: 5 %**

L-IQ- M-IQ H-IQ

L-IQ, M-IQ

L-IQ, H-IQ

M-IQ, H-IQ

**L-IQ = low IQ group; M-IQ = medium IQ group; H-IQ = high IQ group**

2

.755

.378

3.140

.0534

6.279

.565

43

5.172

.120

DF

Sum of Squares

Mean Square

F-Value

P-Value

Lambda

Power

Residual

**Spatial Localization**

**Spatial Misalignment Task**

**ANOVA Table for Hess average**

16

1.198

.344

.086

14

1.165

.361

.097

16

.915

.336

.084

Count

Mean

Std. Dev.

Std. Err.

L-IQ

M-IQ

H-IQ

**Means Table for Spatial Misalignment Task**

0

.2

.4

.6

.8

1

1.2

1.4

Threshold offset (deg)

L-IQ

M-IQ

H-IQ

**Error Bars: ± 1 Standard Error(s)**

.033

.256

.7979

.283

.247

.0259

S

.250

.256

.0552

Mean Diff.

Crit. Diff.

P-Value

**POST-HOC TESTS**

**Fisher's PLSD for Spatial Misalignment Task**

**Significance Level: 5 %**

L-IQ- M-IQ H-IQ

L-IQ, M-IQ

L-IQ, H-IQ

M-IQ, H-IQ

**L-IQ = low IQ group; M-IQ = medium IQ group; H-IQ = high IQ group;**

**S= significant**

2

.837

.418

2.241

.1187

4.481

.420

43

8.030

.187

DF

Sum of Squares

Mean Square

F-Value

P-Value

Lambda

Power

Residual

**Length Discrimination Task**

**ANOVA Table for Length discrimination**

16

.477

.515

.129

14

.566

.502

.134

16

.246

.228

.057

Count

Mean

Std. Dev.

Std. Err.

L-IQ

M-IQ

H-IQ

**Means Table for Length discrimination**

0

.1

.2

.3

.4

.5

.6

.7

.8

Threshold size difference

L-IQ

M-IQ

H-IQ

**Error Bars: ± 1 Standard Error(s)**

-.089

.319

.5778

.232

.308

.1367

.320

.319

.0490

S

Mean Diff.

Crit. Diff.

P-Value

**POST-HOC TESTS**

**Fisher's PLSD for Length discrimination**

**Significance Level: 5 %**

L-IQ, M-IQ

L-IQ, H-IQ

M-IQ, H-IQ

**L-IQ = low IQ group; M-IQ = medium IQ group; H-IQ = high IQ group;**

**S= significant**

L-IQ- M-IQ H-IQ

2

12.237

6.118

2.821

.0706

5.643

.516

43

93.249

2.169

DF

Sum of Squares

Mean Square

F-Value

P-Value

Lambda

Power

Residual

**Shifting Attention**

**Attentive Tracking Task**

**ANOVA Table for Attentive tracking**

16

6.667

1.529

.382

14

6.911

.984

.263

16

7.844

1.744

.436

Count

Mean

Std. Dev.

Std. Err.

L-IQ

M-IQ

H-IQ

**Means Table for Attentive tracking**

0

1

2

3

4

5

6

7

8

9

Total ball number

L-IQ

M-IQ

H-IQ

**Error Bars: ± 1 Standard Error(s)**

-.244

1.087

.6532

-1.177

1.050

.0289

S

-.933

1.087

.0906

Mean Diff.

Crit. Diff.

P-Value

**POST-HOC TESTS**

**Fisher's PLSD for Attentive tracking**

**Significance Level: 5 %**

L-IQ, M-IQ

L-IQ, H-IQ

M-IQ, H-IQ

**L-IQ = low IQ group; M-IQ = medium IQ group; H-IQ = high IQ group;**

**S= significant**

L-IQ- M-IQ H-IQ

2

.005

.003

1.624

.2090

3.248

.313

43

.072

.002

DF

Sum of Squares

Mean Square

F-Value

P-Value

Lambda

Power

Residual

**Ventral Stream Function**

**Hue Discrimination Task**

**ANOVA Table for Hue Discrimination Task**

16

.022

.048

.012

14

.037

.052

.014

16

.010

.011

.003

Count

Mean

Std. Dev.

Std. Err.

L-IQ

M-IQ

H-IQ

**Means Table for Hue Discrimination Task**

0

.01

.02

.03

.04

.05

.06

Hue Difference (%)

L-IQ

M-IQ

H-IQ

**Error Bars: ± 1 Standard Error(s)**

-.014

.030

.3405

.013

.029

.3903

.027

.030

.0785

Mean Diff.

Crit. Diff.

P-Value

**POST-HOC TESTS**

**Fisher's PLSD for Hue Discrimination Task**

**Significance Level: 5 %**

L-IQ- M-IQ H-IQ

**L-IQ = low IQ group; M-IQ = medium IQ group; H-IQ = high IQ group**

L-IQ, M-IQ

L-IQ, H-IQ

M-IQ, H-IQ
